# Intelligent Health Care: Applications of Deep Learning in Computational Medicine

**DOI:** 10.3389/fgene.2021.607471

**Published:** 2021-04-12

**Authors:** Sijie Yang, Fei Zhu, Xinghong Ling, Quan Liu, Peiyao Zhao

**Affiliations:** ^1^School of Computer Science and Technology, Soochow University, Suzhou, China; ^2^WenZheng College of Soochow University, Suzhou, China

**Keywords:** deep learning, computational medicine, health care, medical imaging, genomics, electronic health records, drug development

## Abstract

With the progress of medical technology, biomedical field ushered in the era of big data, based on which and driven by artificial intelligence technology, computational medicine has emerged. People need to extract the effective information contained in these big biomedical data to promote the development of precision medicine. Traditionally, the machine learning methods are used to dig out biomedical data to find the features from data, which generally rely on feature engineering and domain knowledge of experts, requiring tremendous time and human resources. Different from traditional approaches, deep learning, as a cutting-edge machine learning branch, can automatically learn complex and robust feature from raw data without the need for feature engineering. The applications of deep learning in medical image, electronic health record, genomics, and drug development are studied, where the suggestion is that deep learning has obvious advantage in making full use of biomedical data and improving medical health level. Deep learning plays an increasingly important role in the field of medical health and has a broad prospect of application. However, the problems and challenges of deep learning in computational medical health still exist, including insufficient data, interpretability, data privacy, and heterogeneity. Analysis and discussion on these problems provide a reference to improve the application of deep learning in medical health.

## Introduction

In recent years, with the explosive growth of biomedical data and the rapid development of medical technology and computer technology, the field of medical health ushered in the era of big data (Miotto et al., 2018). In this context, computational medicine began to appear as a new subject. Based on big biomedical data and computer technology, computational medicine is an interdisciplinary subject combining medicine, computer science, biology, mathematics, etc. It uses the method of artificial intelligence to intelligently understand the principle and physiological mechanism of human diseases by analyzing big data and provides useful information and guidance for disease prediction, clinical diagnosis, and medical services. Taking the pharmaceutical industry as an example, traditionally, new drug research suffers from long periods, considerable investment, and high failure rate. In contrast, the research based on computational medicine can complete the preclinical drug research and development in an average of 1–2 years, with high success rate and low resource consumption indicating that the field of medical health is gradually entering the era of intelligence and digitization.

However, biomedical datasets are high-dimensional, jumbled, noisy, and sparse, making it difficult to mine the rich information behind these datasets effectively. Therefore, an appropriate approach is needed to process large amounts of biomedical data to obtain efficient information. At present, in the community of machine learning, deep learning is a bright pearl in the field of artificial intelligence. As a branch of machine learning, it has been proved that deep learning is an effective method and surpasses the traditional machine learning in areas such as computer vision (He et al., 2016), natural language processing (Lan et al., 2020), and speech recognition (Abdel-Hamid et al., 2014). The key step of the machine learning method, called feature engineering, is to artificially use expert and domain knowledge to distill features from data and further analyze the features by machine learning models (such as support vector machine, random forest, etc.). In the era of big data, the manual extraction is insufficient and biased such that it cannot establish a high-performance model for specific tasks.

Unlike traditional machine learning approaches, deep learning spares the need to extract features manually, which improves time and resource efficiency. Deep learning is implemented by neural networks consisting of neurons. Each layer of neural networks is composed of a large number of neurons, and the output of the upper layer is regarded as the input of the next layer. Through the connection between layers and the nonlinear processing method, the neural network can convert the original input to the output. More importantly, the high-level network can automatically learn more abstract and generalized features from the data, which overcomes the shortcoming that machine learning needs to extract features manually.

**FIGURE 1:**
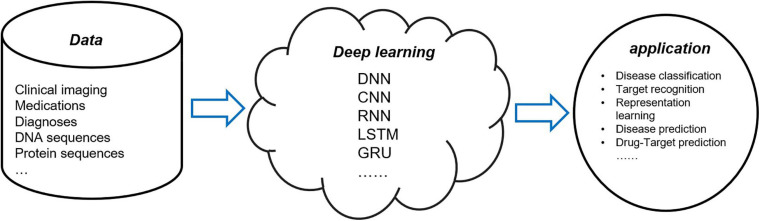
Application of deep learning models in computational medicine.

As the most advanced artificial intelligence method, deep learning provides a method for computational medicine, so it is a trend to apply deep learning method to biomedical data analysis. Figure 1 is a schematic diagram of deep learning in computational medicine. However, biomedical data are not as clean, easy to process, and easy to obtain as data in other fields, so it is a challenge to give a full play to the role of deep learning in computational medicine.

In this article, we first introduce several popular deep learning frameworks. Second, we survey the application of deep learning in clinical imaging, electronic health records, genomics, and drug development. Finally, we point out that the application of deep learning in the field of medical and health faces challenges such as insufficient data, model interpretability, data privacy, and heterogeneity.

## Introduction to Deep Learning

**FIGURE 2:**
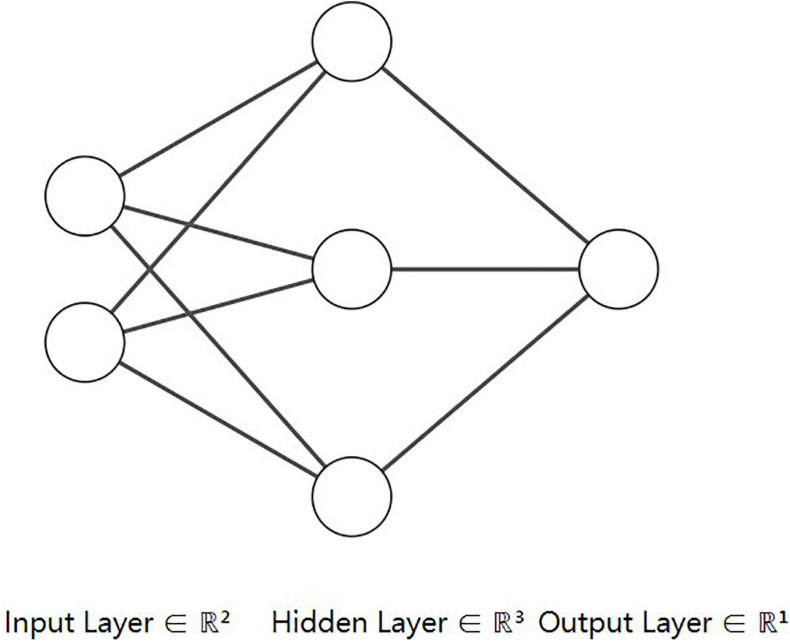
Illustration of neural network architecture.

Deep learning is a part of machine learning, which is inspired by neurons in the human brain: there are tens of millions of neurons in the human brain, and there are more than 100,000 connections between them. The deep learning method is called artificial neural network. As shown in Figure 2, the neural network is composed of the input layer, hidden layer, and output layer. Each layer is composed of several neurons, and the hidden layer may consist of many layers. According to different task types, there are different numbers of neurons in the output layer of the neural networks. For example, there are three neurons in the output layer in the three-classification problem, and each neuron represents the probability of belonging to a certain category.

**FIGURE 3:**
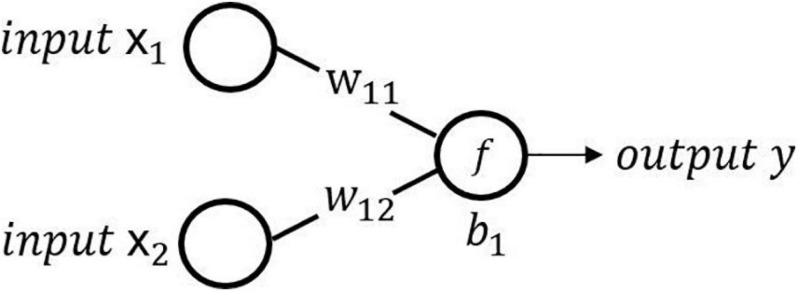
Schematic diagram of neural network calculation.

We take a neuron in the hidden layer as an example to illustrate the calculation method of the neuron. As shown in Figure 3, the calculation of the neuron is as follows:

The equation of the calculation of the neuron is as follows:

(1) y=f⁢(∑jwi⁢j⁢xj*)

where *x*_*j*_ is the *j*th input of the neuron, *y* is the output of the neuron, *w*_*ij*_ is the weight of the *i*th neuron and the *j*th input, *b*_*i*_ is the bias of the *i*th neuron, and *f* is the activation function to perform a nonlinear transformation on the output of the neuron. The common activation functions are sigmoid, ReLU, tanh, softmax, etc.

The training of the neural network depends on forward propagation algorithm and back-propagation algorithm. Forward propagation refers to the whole process of data propagation from the input layer to the output layer, where the neural network calculates intermediate variables of each neuron in turn. Back propagation refers to the parameter optimization process of the neural network. According to the intermediate variables calculated by forward propagation, the parameters of the neural network are updated by gradient descent. The common gradient descent algorithms include random gradient descent, Adam (Kingma and Ba, 2015), RMSprop, Adadelta (Zeiler, 2012), Adagrad (Duchi et al., 2011), etc.

According to the connection and calculation methods, there are different types of the neural networks. Here, we discuss only the most common and basic neural networks, which constitute the basic deep learning methods. Table 1 lists a summary of the neural networks.

**TABLE 1:** A summary of the neural networks.

**Neural networks**	**Advantages**	**Disadvantages**	**Biomedical tasks**
Fully connected neural network	It is widely used at the end of the other neural network models to integrate features and make predictions	It is not easy to process high-dimensional data	Combined with other neural networks, it is widely used in many fields
Convolutional neural network	It can extract highly abstract and complex features from images	It has too many parameters, and the training speed is slow	It is suitable for processing imaging-related tasks, such as clinical imaging
Recurrent neural network	It has a memory function and can effectively process data about sequence and time	Training procedure is difficult and computationally intensive	It is suitable for processing sequence related biomedical data, such as DNA sequence, protein sequence, electronic health records
Autoencoder	It can perform unsupervised learning without using labeled data	It needs a pretraining phase	It is suitable for feature dimensionality reduction or learning effective features from data, such as clinical imaging and genomics
Deep belief network	It can be used for both supervised learning and unsupervised learning	The training process is computationally intensive	It is suitable for automatic feature extraction tasks, such as genomics and drug development
			

### Fully Connected Neural Network

**FIGURE 4:**
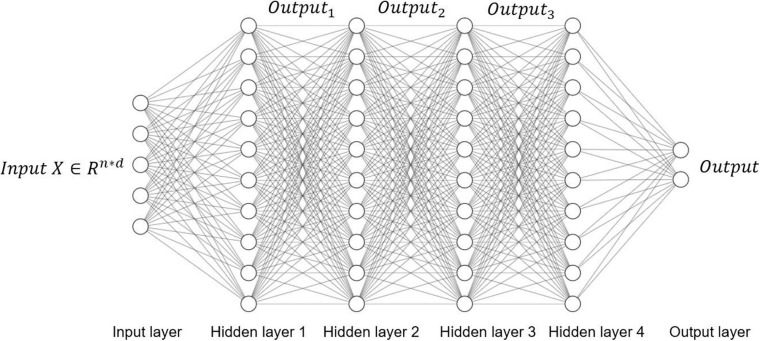
Fully connected neural network.

As the name implies, a fully connected neural network means that the neurons in the layers of the neural networks are completely connected, as shown in Figure 4. The fully connected neural network consists of the input layer, the hidden layer, and the output layer. The input layer is responsible for receiving input data. The hidden layer is composed of many neural network layers for feature extraction. The output layer outputs the final prediction result.

Assume that the input is _X∈R^n^*d_, where the number of samples is *n*, and each sample consists of *d* features. Assume that the first hidden layer *Hidden layer 1* contains *h* neurons, that is, the *Hidden layer 1* contains *h* outputs, and then the weight matrix of the first hidden layer is denoted as _W_1 ∈R^d^*h_, the bias is denoted as _b_1 ∈R^1^*h_, and the calculation of output *Output*_1_ is _*Output_1 =f(XW_1 +b_1)*_.

After that, the output *Output*_1_ of *Hidden layer 1* is used as the input of *Hidden layer 2*, and so on, until the output layer of the fully connected network outputs the final calculation result. The fully connected neural network is the most basic neural network. Combined with other neural networks, it is widely used to integrate high-level features and output prediction results. Depending on the task type, the final output can be either a probability distribution or a task-related value.

### Convolutional Neural Network

**FIGURE 5:**
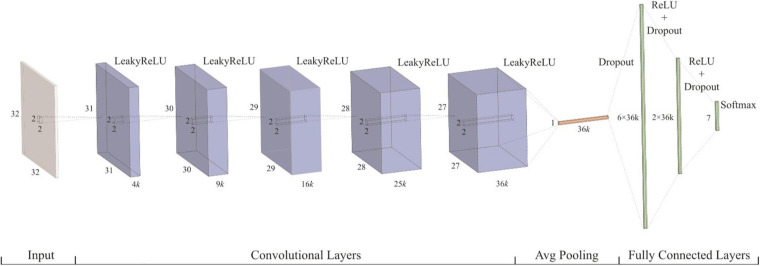
Convolutional neural network.

In recent years, the convolutional neural network has made remarkable achievements in image recognition. The convolutional neural networks adopt the method of local connection and weight sharing, which reduces the complexity of the network and enables the network to directly use the image as input. The convolution neural network has two important characteristics: first, the features learned from the image are translational and nondeformable; second, the higher the convolution layer, the more abstract and complex the features extracted. The convolution neural network is composed of the convolution layer, the pooling layer, and the fully connected layer. The convolution layer is composed of filters. Each filter is equivalent to a small window. These small windows move on the image to learn features from the image. Then, the learned features are subsampled by pooling operation to extract more representative features and improve the robustness and accuracy of the model. Finally, the fully connected layer outputs the prediction result. A convolutional neural network framework for lung pattern recognition (Anthimopoulos et al., 2016) is shown in Figure 5.

### Recurrent Neural Network

Another common neural network is called the recurrent neural network, which is very suitable for processing sequential data, such as time-dependent data.

**FIGURE 6:**
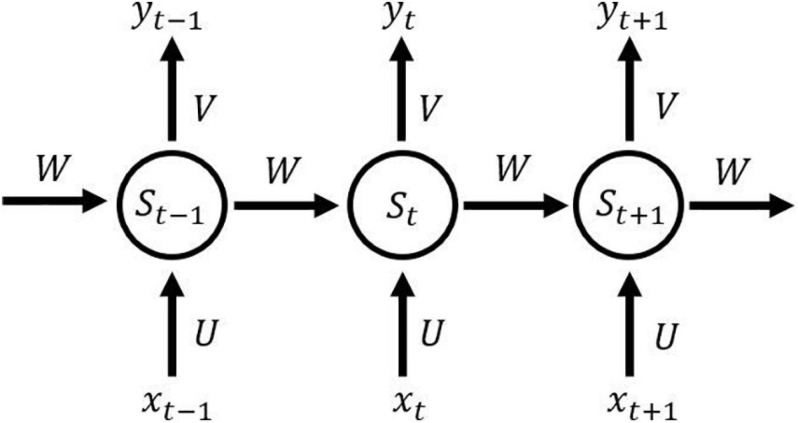
Illustration of recurrent neural network.

In the fully connected neural networks and the convolutional neural networks, their inputs are independent. In contrast, in the recurrent neural network, the former input and the latter input are dependent and have sequence relation. Just like analyzing a sentence, because the current word depends on the front and back words, it means that analyzing each independent word will not produce good results. The structure of the recurrent neural network is shown in Figure 6. At time *t*, the input of the neural network is *x*_*t*_. The output of the neural network is *y*_*t*_, which is calculated from the hidden layer state *s*_*t*_ that depends not only on the input *x*_*t*_ at the current time *t*, but also on the state *s*_*t–1*_ at the time *t*-1, which makes the recurrent neural network have memory, and the state of the last moment can affect the effectiveness of the current time.

The variants of recurrent neural networks include Gated Recurrent Unit (GRU) (Chung et al., 2014) and Long Short-Term Memory (LSTM) (Hochreiter and Schmidhuber, 1997), and so on.

### Autoencoder

The fourth deep learning framework is called the autoencoder, which is often used in unsupervised learning.

**FIGURE 7:**
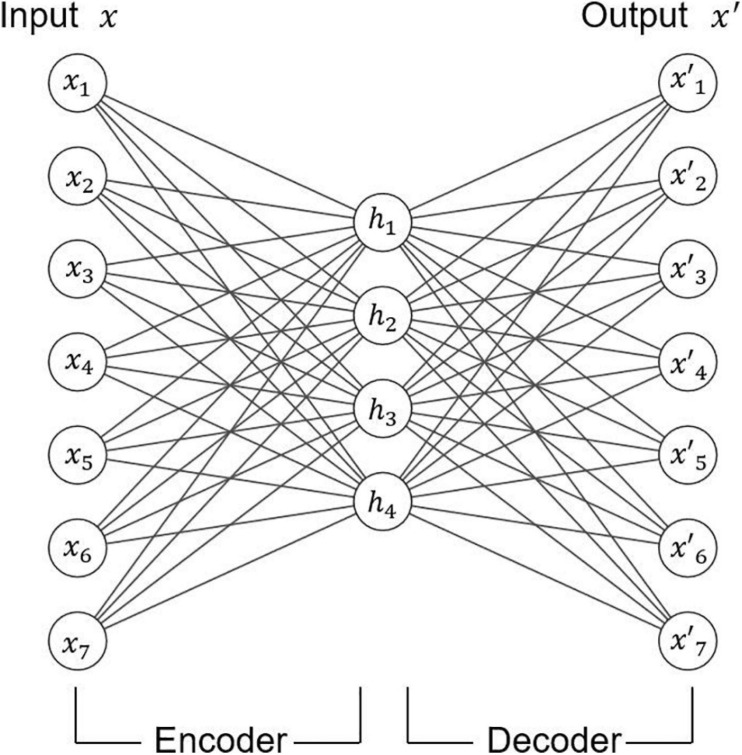
Illustration of autoencoder.

The autoencoder can be used to reduce dimension and learn feature. The structure of the autoencoder is shown in Figure 7. The autoencoder is composed of an encoder and a decoder. Encoders and decoders can be any neural networks models. In general, the number of neurons in the middle-hidden layer is less than that in the input layer and the output layer, which is useful for compressing data and learning effective features from data. The number of neurons in the input layer and the output layer in the autoencoder is the same. Specifically, the encoder reduces the dimension of the original data to get a new representation. Then, the decoder restores the input data through this new representation.

The deformation of autoencoder includes stacked autoencoder (Bengio et al., 2006), denoising autoencoder (Vincent et al., 2008), variational autoencoder, etc. The stacked autoencoder is a hierarchical deep neural network structure composed of multilayer autoencoders. It has deeper depth and stronger learning ability. The denoising autoencoder adds random noise to the input data and then uses the data with noise to train the autoencoder. The autoencoder trained in this way is stronger and has better antinoise ability. Variational autoencoder adds some restrictions in the encoding process, which makes the generated vectors follow the standard normal distribution. The encoding method makes the automatic encoder more effective.

### Deep Belief Network

Deep belief network (Hinton et al., 2006) is a probability generation model based on the restricted Boltzmann machine, which establishes a joint probability distribution between data and label.

**FIGURE 8:**
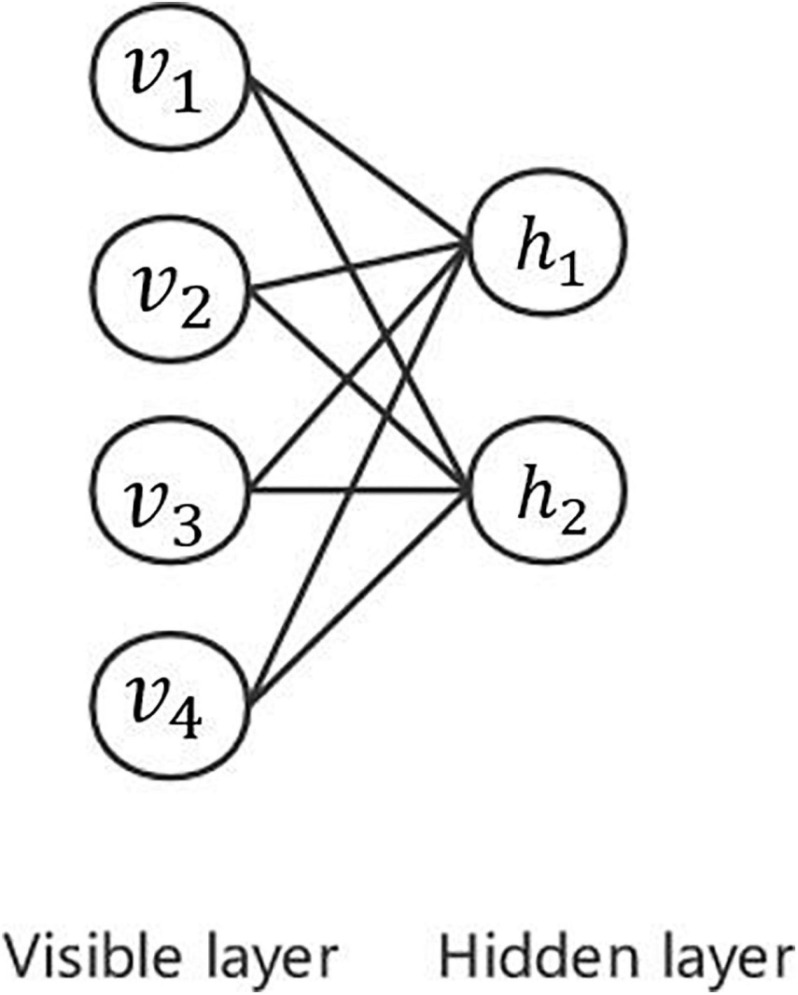
Illustration of restricted Boltzmann machine.

As shown in Figure 8, the restricted Boltzmann machine has only two layers: the visible layer composed of visible units and the hidden layer composed of hidden units. The visible layer is used for the input of training data, whereas the hidden layer is used as a feature detector. Each layer can be represented as a vector, each dimension by each neuron. Neurons are independent of each other. The advantage of this is that given the values of all the explicit elements, the values of each implicit element are independent of each other. The trained restricted Boltzmann machine can extract the features of the explicit layer more accurately or restore the explicit layer according to the features represented by the implicit layer.

**FIGURE 9:**
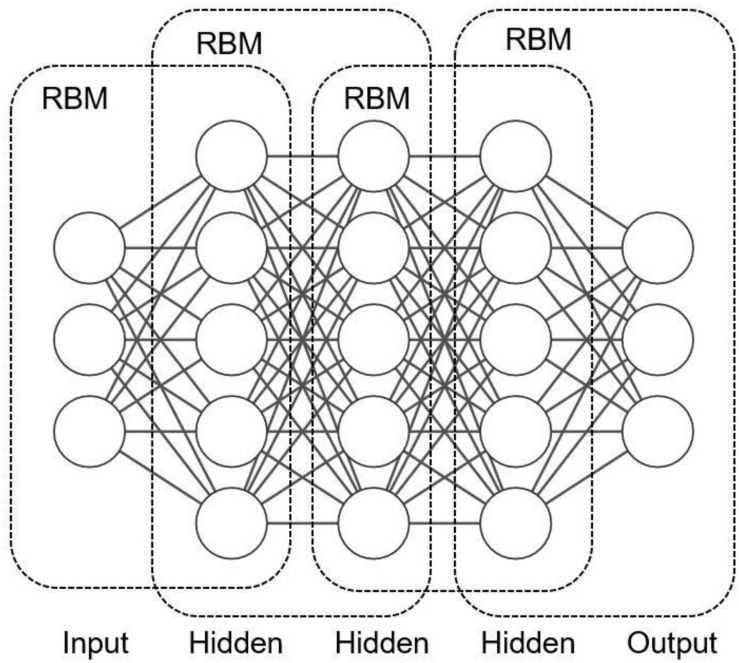
Illustration of deep belief network.

As shown in Figure 9, several restricted Boltzmann machines are connected to form a deep belief network in which the hidden layer of the previously restricted Boltzmann machine is the visible layer of the next restricted Boltzmann machine. It means that the output of the previously restricted Boltzmann machine is the input of the next restricted Boltzmann machine. In the training process, it is necessary to fully train the restricted Boltzmann machine in the upper layer before training the restricted Boltzmann machine in the current layer. The procedure continues until the last layer.

### Software/Hardware Support

Owing to the explosion of data and the support of GPU hardware acceleration technology, deep learning has developed rapidly in recent years. At present, there are many deep learning libraries such as PyTorch, Keras, TensorFlow, Theano, Caffe, and so on. Table 2 lists the representative libraries and packages that provide a convenient and efficient tool for researchers who need to develop deep learning programs and greatly promote the application and development of deep learning in various fields (including computational medicine).

**TABLE 2:** Some frequently used deep learning packages.

**Name**	**Interface**	**URL**
Keras	Python	https://keras.io/
PyTorch	Python	https://pytorch.org/
TensorFlow	Python	https://www.tensorflow.org/
Caffe	C++/Python/MATLAB	https://caffe.berkeleyvision.org/
Theano	Python	http://deeplearning.net/software/theano/
Torch	LuaJIT/C	http://torch.ch/

## Application of Deep Learning in Computational Medicine

### Search Strategy and Selection Criteria

Because Google Scholar provides researchers with a convenient and quick way to search the literature, we searched Google Scholar for published researches from 2015 to 2020 that contained the keyword “deep learning,” which combines the terms of corresponding fields for deep learning in each different application field until September 2020. All searched researches are published in English. Specifically, for the application of deep learning in the clinical imaging, the combination of “deep learning” and “medical image” was used to search. For the application of deep learning in the field of the electronic medical records, the combination of “deep learning” and “electronic health record” or “electronic medical record” was used to search. For the research of deep learning in genomics, the combination of “deep learning” and “genomics” or “gene” was used to search. For the research of deep learning in drug development, we used the combination of “deep learning” and “drug development” or “drug repositioning” or “drug repurposing.” For the literature found, we also conducted manual screening to check whether the content of the article is about the application of deep learning in computational medicine. Finally, this review includes 107 articles.

### Medical Image

The medical image plays a key role in medical diagnosis and treatment providing an important basis for understanding a patient’s disease and helping physicians make decisions. As medical devices become more advanced, and the career of medical health is rapidly growing, more and more medical image data are generated, such as magnetic resonance imaging, computed tomography (CT), and so on. Huge amounts of medical imaging data require much more time if experts analyzed the data alone. And the analysis of medical image data may produce erroneous or biased results due to varying degrees of experience, knowledge, and other factors that the experts themselves have. Machine learning algorithms are, to some extent, able to assist specialists in automated analysis, but may not have the ability to process and achieve high accuracy when faced with such vast data and complex problems.

Deep learning has been successful in the field of image processing: it carries out tasks such as image classification, target recognition, and target segmentation by analyzing images. Therefore, the application of deep learning in the task of medical image analysis has become a trend in medical research in recent years. Researchers used the artificial intelligence methods to help physicians make accurate diagnoses and decisions. Many aspects are involved in these tasks, such as detecting retinopathy, bone age, skin cancer identification, etc. Deep learning achieves expert level in these tasks. The convolutional neural network is a powerful deep learning method. The convolutional neural networks follow the principle of translational invariance and parameter sharing, which is very suitable for automatically extracting image features from the original image.

**FIGURE 10:**
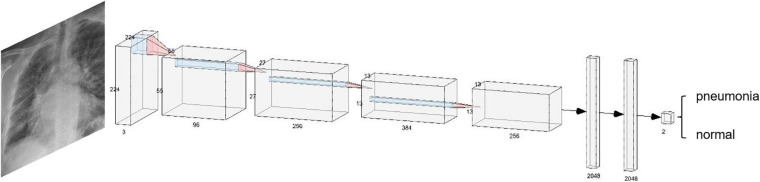
Convolutional neural network structure for detecting pneumonia with chest X-ray images.

Figure 10 shows a convolutional neural network structure for detecting pneumonia with chest X-ray images. The convolution neural network is composed of the convolution layer, the pooling layer, and the fully connected layer. First, the filtering window in a convolution layer will move step by step in the image to learn local features from the image. Second, the pooling layer will sample the learned features to reduce the parameters and overfitting to improve the performance of the network. Finally, these features will output the final results through the fully connected layer.

In a convolution neural network, there are usually multiple convolution layers such that the convolution layer at the bottom can learn the local features in the image, and the high-level convolution layer can integrate these local features and learn the overall features. Usually, for the same symptom, the location and shape of lesions or tumors are different in different pictures, making it very difficult to analyze. The advantage of the convolutional neural network is that it can automatically learn local features from images and integrate them into global features. Therefore, the convolutional neural network is very suitable for clinical image processing.

Researchers have analyzed the effects of different structural convolutional neural network applications on clinical images. Shin et al. (2016) pointed out that even if the available training dataset is limited, a convolutional neural network architecture with a depth of 8 or even 22 layers may be useful. They also proved that it was beneficial to migrate models trained from large-scale annotated natural image datasets (ImageNet) (Russakovsky et al., 2015) to computer-aided diagnosis problems in experiments. It can be seen that the convolutional neural network with few layers can obtain satisfactory results in limited datasets and has great potential in clinical imaging.

Many researchers have used convolutional neural networks on the task of the fundus image and achieved good results (Grinsven et al., 2016; Gulshan et al., 2016; Dai et al., 2018). Poplin et al. (2018) predicted cardiovascular risk factors from retinal fundus images. In order to better understand how neural network models can predict, a deep learning technology called “soft attention” is used, which can identify the parts that affect the prediction of the model. Kermany et al. (2018) developed a deep learning system to effectively classify the images of macular degeneration and diabetic retinopathy. Fauw et al. (2018) used a three-dimensional U-Net (Ronneberger et al., 2015; Çiçek et al., 2016) architecture to segment the original optical coherence tomography images into 15 types of tissue maps. Experimental results showed that the proposed method achieved and even surpassed the performance of experts in referral decision-making and disease prediction.

Other researchers used convolutional neural networks to detect or classify diseases on chest X-ray datasets. For example, Cao et al. (2016) used the convolutional neural network to X-ray images collected from mobile devices to diagnose tuberculosis. The accuracy of the binary classification task reached 89.6%, which proved the learning ability of the convolutional neural network in the medical field. Cicero et al. (2017) used convolutional neural networks to detect and classify chest abnormalities. Yuan et al. (2019) conducted an experiment with collaborative deep learning on chest X-ray images. Through collaborative deep learning, the accuracy was improved by approximately 19%. Liu et al. (2019) proposed a deep fusion network based on segmentation to obtain the features of the whole chest X-ray image and local lung region image from the image. Kleesiek et al. (2016) used convolutional neural networks to analyze the brain from magnetic resonance imaging. The method could deal with any number of patterns, which proved the feasibility of the convolution neural network in large-scale research.

In other clinical image tasks, the convolution neural network also achieved the performance of doctors including skin cancer diagnosis, knee osteoarthritis diagnosis, bone age assessment, etc. (Esteva et al., 2017; Haenssle et al., 2018; Iglovikov et al., 2018; Rakhlin et al., 2018; Tiulpin et al., 2018).

In addition to tasks of routine disease detection, the convolution neural network can also be used to evaluate the operation of doctors to help doctors improve the operation effect. Jin et al. (2018) used a convolutional neural network to automatically evaluate the performance of surgeons by tracking and analyzing tool movements in surgical videos. Shvets et al. (2018) introduced a method of robot instrument segmentation from surgical images based on deep learning. They used four different deep learning frameworks: a modified U-Net, two modified TernausNet (Iglovikov and Shvets, 2018), and a modified LinkNet (Chaurasia and Culurciello, 2017). The modified TernausNet performed best in binary segmentation experiments and partial segmentation experiments.

Some researchers also use data augmentation technology to solve the problem of data sparsity to prevent model overfitting (Anthimopoulos et al., 2016; Avendi et al., 2016; Kooi et al., 2017). Data augmentation refers to using some methods such as flipping, rotation, translation, clipping, changing contrast to transform the original image to generate more training data from the existing training samples, solving the problem of insufficient data, and improving the ability of the model.

In addition to the convolution neural networks, other neural networks methods such as the autoencoder, the deformation model of autoencoder, and the recurrent neural network are also used in medical imaging research. For example, Hu et al. (2016) constructed an autoencoder architecture to classify patients and predict Alzheimer disease. Compared with the support vector machine, the prediction accuracy is improved by approximately 25%. Cheng J. Z. et al., 2016) used the stacked denoising autoencoder structure for the differential diagnosis of breast lesions in ultrasound images and pulmonary nodules. Mansoor et al. (2016) used the stacked autoencoder to locate anterior visual pathway segmentation and to create a model to capture local appearance features of anterior visual pathway segmentation. Ortiz et al. (2016) used the set of deep belief networks for early detection of Alzheimer disease. Andermatt et al. (2016) proposed multidimensional GRU for brain segmentation, which could accurately segment three-dimensional data.

In conclusion, in the field of clinical imaging, the most used deep learning network is convolutional neural networks, other neural networks such as recurrent neural networks and autoencoder have also been used. To make the models capture the patterns and features, some measures have also been taken by researchers, such as regularization, dropout, and expansion of the dataset methods. Among them, the most common approach for expanding dataset is data augmentation techniques, which play an important role in improving the performance of models. These experiments demonstrate that deep learning performs better than traditional machine learning on clinical imaging tasks. Deep learning provides doctors with automated technology to analyze pictures, videos, etc., to help biomedical careers develop rapidly.

Although the effect of deep learning on medical imaging is better than that of machine learning, and deep learning achieves human-level performance, there are still some limitations:

(1) It is difficult to collect sufficient labeled data. Training a convolutional neural network with good performance requires a large number of parameters and samples. Sometimes it is difficult to collect sufficient and labeled training data. In addition to the differences in features, patterns, colors, values, and shapes in real medical image data, it is difficult to train a suitable network.
  There are two ways to deal with the problem. The first way is the strategy of data augmentation, which is a very powerful technology to reduce overfitting. It generates a new image through a series of transformations (such as translation, flipping, changing contrast) of the original images to expand the dataset, so that the model has better generalization ability. The other is to use transfer learning, which uses a model trained in other training data in advance and then transfers the model to the medical image data to fine-tune the model, so as to get a model with strong generalization ability.

(2) Convolution neural networks cannot explain the hierarchical and positional relationship between features extracted from images. For example, neurons can capture the dataset feature, but neurons cannot well capture the spatial relationship between these features. For this reason, Sabour et al. (2017) proposed the Capsules Network. In this structure, the input and output of the capsules are not a scalar, but a vector instead of a traditional neuron. The length of the vector means the probability of the existence of instances, while the value of the vector can represent the relationship between features.
  At present, there are few types of research on the Capsules Network in medical imaging. Jiménez-Sánchez et al. (2018) have studied the application of Capsules Network in clinical imaging. The experimental results showed that Capsules Network could be trained with fewer data to obtain the same or better performance, and it was more robust for unbalanced class distribution. This result undoubtedly brought new ideas and directions to the application of deep learning in medical imaging.

(3) Convolutional neural networks are suitable for processing two-dimensional image data, but the images produced by magnetic resonance imaging or CT image have the inherent three-dimensional structure. If the convolutional neural network is used to process these medical images, key information will be lost.

### Electronic Health Record

One-dimensional convolutional neural network, recurrent neural network, LSTM, GRU, and other neural networks in deep learning have been widely used in the natural language processing community and have achieved great success. These networks are very suitable for processing sequence-related data, such as sentence, voice, time series, and so on. Similarly, natural language processing technology is also used in the field of computational medicine, which uses these neural networks to process electronic medical records.

In recent years, electronic health record has received more and more attention. Electronic health record stores the treatment information of patients in electronic form. The information includes structured demographic information, diagnosis information, drug information, operation process information, experimental test results, and unstructured clinical text (Jensen et al., 2012). Mining electronic health records can improve the efficiency and quality of diagnosis and promote medical development. For example, it provides timely treatment for patients by mining the data in the electronic health record to predict the disease, or it analyzes the hidden relationship between diseases and diseases, diseases and drugs, and drugs and drugs in the electronic health records to provide help for doctors in decision-making.

In short, for patients, the use of electronic health records can better help patients understand their physical condition and disease status; for the medical staff, the use of electronic health records can help them better analyze problems and provide effective solutions.

Traditionally, machine learning is used to analyze electronic health record data. Usually, we need to extract the features manually and then input them into the model. This feature extraction method often depends on the professional domain knowledge of the extractor, and it may be difficult to find the hidden relationship in the data. Therefore, the quality of the model prediction results is affected by the quality of the manually extracted features. Moreover, this method causes huge human and time loss and affects the research efficiency.

Deep learning overcomes the disadvantage of traditional machine learning, which needs manual feature extraction. However, because of the particularity and complexity of electronic health record data, there are some problems when using deep learning method to deal with them. There are many clinical concepts in the electronic health records, which contain rich information. These concepts are recorded in the form of coding, such as diagnostic coding, disease coding, drug coding, etc. Different medical ontologies formulate the rules of coding and the meanings they represent. At the same time, doctors record these clinical concepts in chronological order. However, it is difficult to find and explore the relationship between these concepts simply by coding the patient’s condition.

**FIGURE 11:**
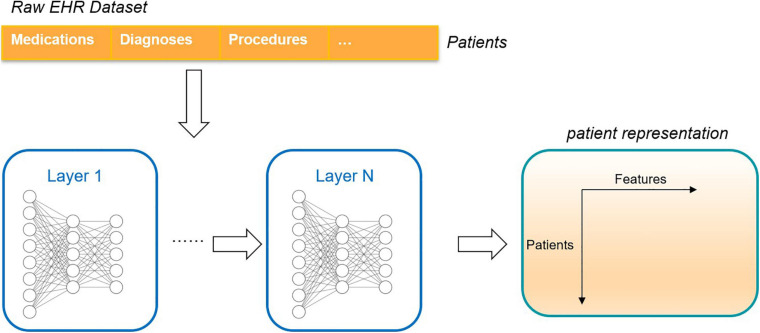
An unsupervised deep learning framework that converts the raw electronic health record into a deep representation of the patient.

The traditional method is one hot coding whose dimension represents the number of medical concepts. The coding method does not reflect the relationship between concepts. Thus, researchers put forward representation learning, which studies how to map the clinical concepts represented by coding in electronic health records into low dimensional space and transform them into low dimensional features (embedding) for representation. At the same time, these low-dimensional features reflect the relationship between different concepts. After getting the representation of clinical concepts, researchers can find out the relationship between them through analysis, such as the relationship between diseases, or diseases and clinical events, or use these concepts in further tasks to provide useful information for doctors and help doctors make decisions. These studies included analysis of mortality, prediction of rehospitalization, prediction of disease, etc. As shown in Figure 11, the electronic health record can be transformed into patient representation by using the deep learning method (Miotto et al., 2016).

Most of the researches using deep learning to represent the clinical concept of electronic health records use the skip-gram structure of the word2vec model (Mikolov et al., 2013). The skip-gram approach assumes that the meaning of a concept depends on its context (or neighbors). Therefore, given the sequence of a concept, the skip-gram method predicts the context of a target concept when it selects it. After getting the representation of clinical concepts, researchers can find out the relationship between them through analysis, such as the relationship between diseases, or diseases and clinical events. Researchers can also use these concepts in further tasks to provide useful information for doctors and help doctors make decisions (Choi Y. et al., 2016; Choi et al., 2016a,b,c).

Attention mechanisms are also used in the analysis of electronic medical records. The mechanism of attention enables deep learning models to focus from the multitude of information to more critical and important information that is more closely connected to the task. Using the attention mechanisms, it is possible to know what information in the data contributes to the model’s predictions. Zhang J. et al., 2018 established a framework by using recurrent neural networks and attention mechanisms to learn the representation of patients from the temporal electronic health record data. Then, they applied the model to the risk prediction task of future hospitalization. The experimental results showed that deep learning model can achieve a more accurate prediction effect.

Several works have shown that using the combination of different neural networks or different methods can improve the accuracy and efficiency of models. For example, Miotto et al. (2016) used an unsupervised deep learning method to learn the patient’s representation from the electronic health records. They used a three-layer stacked denoising autoencoder to capture the hierarchical relationship and dependence between the data. Ma et al. (2018) proposed a deep learning framework, which is composed of recurrent neural networks and convolutional neural networks to extract patient information patterns. Rajkomar et al. (2018) used LSTM, the attention mechanism, and the single-layer decision tree to learn information from the dataset. In all tasks, the performance of the deep learning model is better than that of the traditional clinical prediction model.

In addition to using deep learning to represent clinical concepts in electronic health records, researchers also use deep learning to carry out disease prediction tasks. Disease prediction refers to predicting whether a patient will suffer from a certain disease or find out the factors related to the disease according to the patient’s electronic health record information. Because the electronic health record contains a wealth of patient information, some indicators and characteristics in the information can be used as a reference to predict whether the patient has a disease. Nickerson et al. (2016) explored two neural networks architectures to deal with issues related to postoperative pain management. Pham et al. (2016) introduced a deep dynamic neural network for tasks such as predicting the next stage of illness. The results are competitive compared with other excellent methods at present. Nguyen et al. (2017) introduced a deep learning system to learn how to extract features from the electronic health record and automatically predict future disease risk. Compared with the traditional technology, the system can detect meaningful clinical patterns and reveal the potential structure of the disease and intervention space.

To deal with the missing values in medical records, Che et al. (2017) developed a model that was based on the GRU. The model captures the observations and their dependence by applying masking and time interval methods to the input and network state. Cheng Y. et al., 2016 used convolutional neural networks to extract phenotypes. They verified the validity of the proposed model on real and virtual electronic health records.

It can be seen that as the electronic health record is sequential, recurrent neural networks such as LSTM and GRU have a very wide range of applications in the field of electronic medical records. Recurrent neural networks are well suited for processing electronic medical records and achieve better results than traditional methods. When using deep learning method to mine the information of the electronic health records, most of the methods are the supervised learning methods; that is, the data are labeled. Some researchers use unsupervised learning to study electronic health records. With the deep learning methods, the patterns in the electronic health record are analyzed. Then the learned patterns are used in disease prediction, event prediction, incidence prediction, and other tasks, which is undoubtedly the trend of the application of deep learning in the field of the electronic medical record.

Many studies have proven the effectiveness of deep learning in the electronic health record. However, there are still some challenges that hinder the further application of deep learning in the electronic health record:

(1) There are many types of data in the electronic health record, which are heterogeneous such that it is difficult to use the data in electronic health records in medical applications. There are five types of electronic health record data types: numerical type, such as body mass index; date–time object type, such as patient admission date; category type, such as race and international disease code; text type of natural languages, such as patient discharge summary; and time-series type, such as patient history (Shickel et al., 2018). In addition, the electronic health record also has the characteristics of high dimension, noise, complexity, and sparsity. How to use a suitable model for different types of the electronic health record is a big challenge when using deep learning methods to process electronic health record data.
(2) The coding in the electronic health record is different because of the difference in medical ontology in reality, which also brings challenges to applying of deep learning in the electronic health record. For example, medical ontology has the Unified Medical Language System (Unified Medical Language System, 2031), *International Classification of Diseases, Ninth Revision* (*ICD-9*) (ICD9, 2032), *ICD-10* (ICD10, 2033), National Drug Code, and other codes. In the specific implementation, different regions or different hospitals do not strictly abide by the coding rules of the medical oncology, so there are nonstandard records. And sometimes, the same disease phenotype can be represented by different medical ontology. For example, in the electronic health record, patients diagnosed with “type 2 diabetes mellitus” can be identified by the laboratory value of hemoglobin A_1c_ > 7.0, the code of 250.00 in *ICD-9*, and the writing method of “type 2 diabetes mellitus” in the clinical text (Miotto et al., 2018). The above problems increase the difficulty of data processing. In addition, the mapping between these codes also brings difficulties to researchers.
(3) The information in electronic health records may have a long-time range, which makes the application of deep learning more difficult. It is very difficult to find a wide range of patients in electronic medical records. For such a long time series of information, it is very difficult to confirm the mapping relationship between symptoms and seizures.

### Genomics

Genomics studies the function, structure, editing, and performance of genes. Because of its powerful ability to process data and automatic feature extraction, many researchers have applied it to the field of genomics to discover deeper patterns.

Compared with traditional machine learning methods, the deep learning methods can extract the higher dimensional features, richer information, and more complex structure from biological data. In recent years, deep learning has been widely used in genomics, such as gene expression, gene slicing, RNA measurement, and other tasks. Deep learning brings new methods to bioinformatics and helps to understand the principles of human diseases further.

**FIGURE 12:**
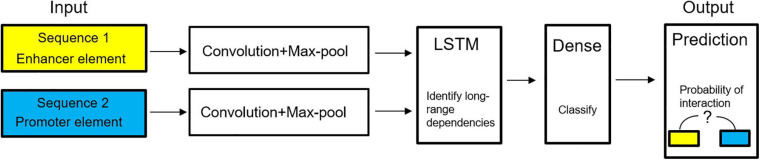
Diagram of deep learning model to predict enhancer–promoter interactions (Chen Y. et al., 2016).

In genomics, deep learning can effectively understand the cause and development process of diseases from the molecular level, and the interaction between genes and environment, and understand the factors leading to disease. The deep learning method can capture the relationship between disease and gene from the high-throughput biological dataset. Doctors can understand the disease more comprehensively, make accurate decisions, and provide patients with more appropriate treatment and diagnosis. The application of deep learning in genomics has greatly promoted the development of personalized therapy and precision medicine. A model predicting enhancer-promoter interactions is shown in Figure 12.

Several studies have demonstrated the effectiveness of deep learning applications on genomics tasks better than traditional machine learning methods. Many researchers have researched the task of gene expression using the deep learning method. For example, Singh et al. (2016) proposed a deep learning model to automatically extract complex histone modifications between essential functions to classify gene expression. Chen Y. et al., 2016 proposed a multitask multilayer neural network to infer the expression of the target gene from the expression of the marker gene. In terms of the average absolute error of all genes, deep learning is better than linear regression, and the performance is improved by 15.33%. Tan et al. (2016) used the gene expression denoising autoencoder to integrate various gene expression data and capture patterns corresponding to biological states. Singh et al. (2017) proposed a deep learning method to discover the interaction between each chromatin marker signal. Badsha et al. (2020) developed the method to understand the dependent structures between genes stored in parameter estimates. Kong and Yu (2018) integrated the external relationship information of gene expression features into the deep neural network. The application of real data proved the practicability of the new model in classification and biological feature selection. Mostavi et al. (2020) proposed three unique convolutional neural network architectures for different data formats. These architectures used high-dimensional gene expression inputs and predicted cancer types while considering their origin tissues.

In addition, other researchers also used deep learning for other genomic tasks, such as predicting the binding sites of RNA-binding proteins (Zhang et al., 2016), predicting DNA methylation status (Angermueller et al., 2017), predicting enhancer–promoter interactions (Singh et al., 2019), and predicting mRNA abundance (Washburn et al., 2019; Agarwal and Shendure, 2020). Besides, Kelley et al. (2016) used the convolutional neural networks to understand the functional activity of DNA sequences from genomic data. Qin and Feng (2017) developed the model to predict cell-specific transcription factor binding based on the available ChIP-seq data. Compared with the existing methods, the model can achieve considerable accuracy in the combination of transcription factors and cell lines with ChIP-seq data. Jha et al. (2017) used an autoencoder to integrate other types of experimental data into the spliced code model for selective splicing. Chen Y. et al. (2016) used an autoencoder to more than 1,000 yeast microarrays to understand the coding system of the yeast transcriptome mechanism and the hierarchical organization of the yeast transcriptome mechanism. Zhou et al. (2018) proposed a deep convolution neural network model that predicts tissue-specific transcriptional effects of mutations, including rare or undetected mutations.

Researchers also used natural language processing technology to represent gene sequence information. Zou et al. (2019) used word embedding technology to represent mRNA subsequences. Then, they used the convolutional neural networks to predict N6-methyladenosine from mRNA sequence.

Some researchers also use the convolutional neural networks for other tasks. Gao et al. (2020) used convolutional neural networks to analyze the global activity of splicing modified compounds and identify new therapeutic targets. Experiments have shown that the deep learning methods could recognize the sequence features and predict the response to drug regulation. Yuan and Bar-Joseph (2019) proposed the convolutional neural network model to infer the relationship between the different expression levels encoded in the genes. In many aspects of performance, convolutional neural network was better than the previous methods.

Researchers have also used several deep learning networks such as convolutional neural networks, recurrent neural networks, autoencoders, and deep belief networks in genomics. For example, in tasks involving DNA sequences and protein sequences, one-dimensional convolutional neural networks and recurrent neural networks are the two most commonly used networks. The one-dimensional convolutional neural network extracts high-level features from the genome sequence data through the movement of the convolution window. The recurrent neural networks can effectively process the information about the sequence and discover specific patterns from the genome sequence data through its memory characteristics. Also, the methods used in natural language processing are also applied to the field of genomics, which provides innovative ideas and methods for the study of genomic data. To make full use of the information in the data, multimodal learning using different data sources is also one of the methods used by researchers. In conclusion, deep learning offers the possibility of mining features from genomic data to help develop a deeper understanding of the disease.

Although deep learning has made impressive achievements in genomics, some problems still exist. The problems of deep learning in genomics are as follows:

(1) Deep learning training usually requires a large number of datasets, and the quality of these datasets is required to be high so that the deep learning model can learn distinguishing features and patterns from the data. However, data insufficiency still exists in genomics, so the model cannot learn from sufficient data and cannot provide key information for researchers or doctors.
  There are many parameters in the deep learning model, and there are a lot of deep learning frameworks. If the amount of data is not enough, it is difficult for researchers to find a deep learning model suitable for the current task. It is easy to see that the model has poor performance in the dataset. Because the deep learning model only remembers the characteristics and patterns of the data in the training set but does not learn the deep relationship between them from the data.
  Generally speaking, there are three measures to solve the problem of insufficient data: The first measure is to expand the dataset from the data layer, such as using data enhancement technology to increase the sample size; the second measure is to use regularization and dropout methods to improve the performance of the deep learning model and increase the generalization ability of the model; the third measure is to use the transfer learning technology to train the model on an unrelated but large number of datasets, and then the trained model is used in the task of their concern, and the parameters of the model are adjusted to get the suitable model. In the above studies, we can see that some researchers have used these techniques to solve the problem of insufficient data in the field of genomics, thus improving the model effect.
(2) Biomedical data are complex and need professional domain knowledge to analyze. Unlike other fields, in genomics, the structure and function of the genome are a very complex model, which puts forward higher requirements for the interpretability of the deep learning model. In recent years, the multimodal learning has become an attempt to improve the interpretability and accuracy of the model. The so-called multimodal learning refers to the combination of data from different input sources and the establishment of different types of deep learning models for different types of data to make full use of the relationship and characteristics of different types of data, so the model can make a more comprehensive and accurate prediction. Many researchers have begun to combine different types of data, such as gene sequences with other types of data such as electronic health records and medical imaging, to expand the field of knowledge and provide more insights for doctors.

### Drug Development

In recent years, with the rapid growth of biomedical data, deep learning technology has become a new method in drug development. The application of deep learning in the field of drug development can help researchers effectively carry out drug development and disease treatment research and greatly promote the development of precision medicine.

Drug development is a complex process. Traditionally, it takes at least 10 years from the development of a new drug to its marketing, which is a very long and resource-consuming process. Traditional drug development is divided into two methods: one is the experimental method, which not only consumes time and efficiency but also causes huge cost; the other is the computational method, which can save time and reduce loss.

**FIGURE 13:**
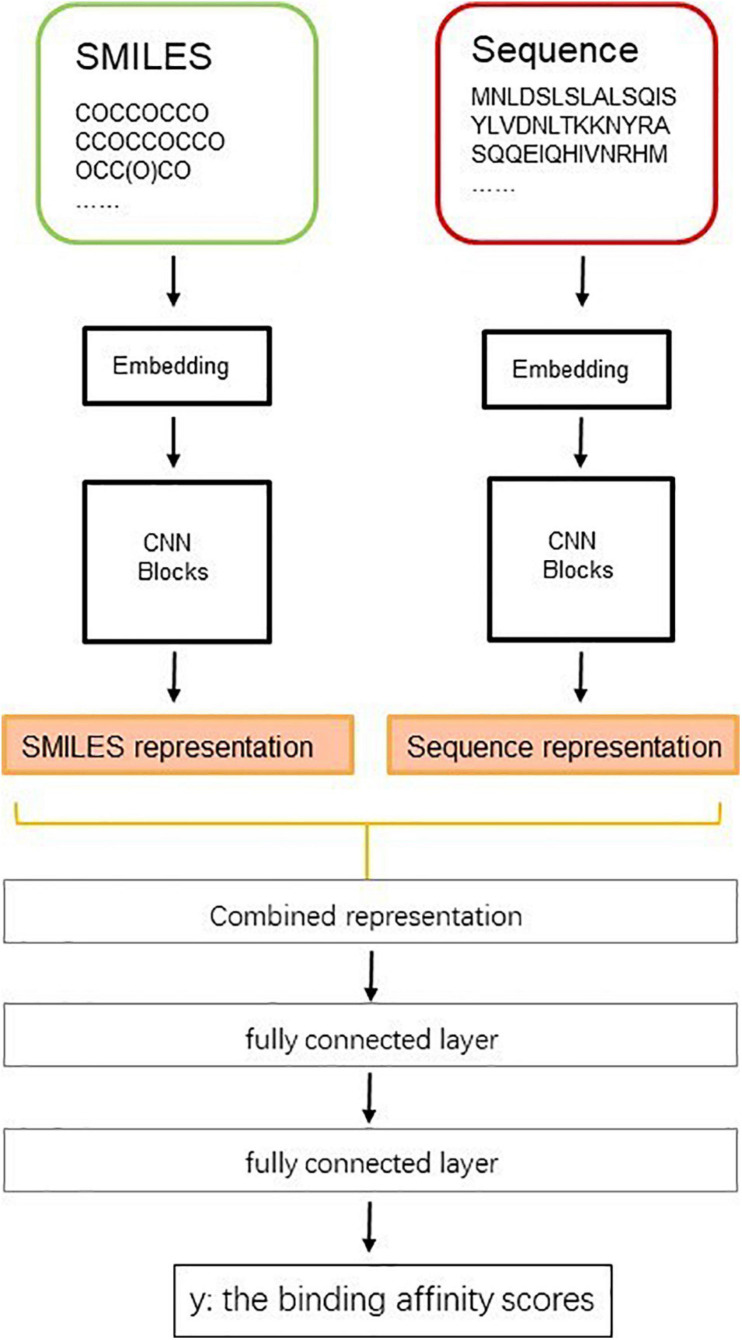
Diagram of deep learning model to predict the binding affinity scores of drugs–targets.

In drug development and design, identifying the interaction between drug and target is an important step. This step can save resources and accelerate the time from drug development to market. In recent years, the biomedical data have increased significantly, which provides a data basis for applying deep learning in drug development. Many researchers have begun to apply deep learning to explore the relationship between drugs and targets. Figure 13 shows a diagram of a deep learning model predicting the binding affinity scores of drug–targets.

Many researchers have begun to apply deep learning to explore the relationship between drugs and targets. They have achieved good results, demonstrating the great ability of deep learning in the field of drug development. For example, Wen et al. (2017) used deep belief networks to predict the interaction between drugs and targets. The model automatically extracted the characteristics of drugs and targets from the simple chemical substructure and sequence information in an unsupervised way. Wang et al. (2017) predicted potential unknown drug–target interactions from drug molecular structures and protein sequences. They used a stacked autoencoder to learn useful information of protein sequences automatically. Öztürk et al. (2018) used two convolutional neural network frameworks to learn features from protein sequences and compounds’ SMILES strings and then combined the learned features into a fully connected layer to predict the interaction between drugs and targets binding affinity. Zong et al. (2019) used the deep learning methods to calculate the vertex similarity and then input the similarity into two rule-based inference methods. Hu S. et al., 2019 represented each drug target pair by connecting the coding vector of the target descriptor and the drug descriptor. Then, they inputted the representations into the convolutional neural network. They evaluated the ability of the model on the DrugBank dataset, with an accuracy of 0.88 and an area under the curve value of 0.95. The results show that the model can be used to distinguish drug and target interactions.

Other investigators have also made efforts to understand drug–target interaction. Hu P. et al., 2019 used the autoencoder with a cascade structure to obtain the deep expression of the fusion network and identify the drug–target interaction. Wang et al. (2020) combined evolutionary characteristics of proteins with drug molecular structure fingerprints to form the feature vector of drug–target pairs. Then, they used LSTM on the feature space to predict the interaction between drug and target. Zeng et al. (2020) developed a deep learning model for target recognition and drug reuse by learning low dimensional vector representations of drugs and targets. Zhao et al. (2020) used two generative adversarial networks to calculate binding affinity between drug and target in an unsupervised way. Then, a convolution neural network is used for prediction. The experimental results show that the proposed method can make full use of the unlabeled data and obtain competitive performance.

In addition to using the deep learning method to predict the interaction between drugs and targets, some researchers also use deep learning to design molecules from scratch, predict the pharmacological properties and synergistic effects of drugs, etc. For example, Aliper et al. (2016) proposed the deep learning model to map transcriptome data to therapeutic categories. In the experiments, the model achieved high classification accuracy and is better than the support vector machine model. Gupta et al. (2018) used the deep learning method to design molecular weight, which captured the semantics of molecular representation and carried out a virtual compound design. Preuer et al. (2018) proposed DeepSynergy to predict the synergistic effect of anticancer drugs. Experiments showed that the model can explore the synergistic effect of drugs and novel combinations in cell line space with high precision. Zhang X. et al., 2018 learned the representation of each molecule in an unsupervised way. Zeng et al. (2019) developed a model that learns advanced features of drugs from different networks through a multimode autoencoder.

Representation learning techniques also have applications in drug development. By combining unsupervised representation learning with deep learning techniques, Wan et al. (2019) used the word2vec to learn the low dimensional representation of compounds and proteins to predict the interaction between unstructured compounds and proteins. Zhang et al. (2020) used deep learning methods to represent molecules as vectors to identify potential drugs, peptides, or small ligands targeting protein targets in the 2019-nCoV virus. The model had high speed and high accuracy, which was suitable for screening thousands of drugs in a short time in some emergencies. Karimi et al. (2020) developed the deep generation model for drug combination design. They used hierarchical variational graph autoencoders to jointly embed gene–gene, gene–disease, and disease–disease networks.

As one of the state-of-the-art methods in artificial intelligence, deep learning provides an important opportunity for drug development. It not only saves drug development time and human resources but also enables the efficient mining of information that is difficult for people. Although modern medical careers progress rapidly nowadays, there are still diseases for which it is still difficult to find drugs to treat them. The deep learning method for drug development has provided a possible breakthrough in finding drugs to treat these diseases. It can be seen that in drug development, researchers used convolutional neural networks, recurrent neural networks, autoencoders, and fully connected neural networks. To deal with the small labeled datasets, there are also studies using unsupervised learning methods for application in the field of drug development. For example, several researches use unsupervised methods to predict the interaction of drug targets and predict the binding affinity of drug targets. In unsupervised learning, autoencoders and their variants, generative adversarial networks, and deep belief networks are commonly used neural networks. They can recover data well or find advanced features from the data for the deeper application.

Although deep learning has broad prospects in drug development, limitations still exist:

(1) It is known that deep learning requires a lot of labeled data. But in the field of drug development, the labeled data are limited. At present, many researchers use semisupervised learning or unsupervised learning to find key information from unlabeled biomedical data. However, even if semisupervised learning or unsupervised learning is used, it is difficult to find useful information in these unlabeled data.
(2) For deep learning, it is difficult to scientifically explain the reasons for making predictions because the occurrence and process of disease are a very complex biomedical field. Deep learning is considered as a “black box” method. If deep learning cannot provide a good explanation, it will be difficult for doctors to believe the prediction results given by deep learning, so they cannot make decisions. It is possible to integrate other types of data and information into drug development to solve the problem of model interpretability.

### Deep Learning Research on Longitudinal Datasets

In this section, we introduce the application of deep learning in longitudinal datasets. Longitudinal data track and record the patient’s long-term condition. Using longitudinal datasets, we can perform tasks such as predicting disease-related risks, predicting the trajectory of relevant biomarkers at different disease development stages, or conducting survival analysis. Some longitudinal data studies, such as Framingham Heart Study (Araki et al., 2016) or the UK Cystic Fibrosis Registry (Taylor-Robinson et al., 2018), provide useful datasets for longitudinal data research.

The patient’s medical history may contain some information about the future disease, so it is very useful to study the history and infer the future disease development from the past information. It requires doctors to make a prediction as soon as possible so that doctors can take measures to prevent the trend of disease onset or deterioration. However, the use of longitudinal data for research will also have difficulties. For example, for predicting disease trajectory, for a patient, his disease status may develop slowly, which increases the difficulty of related research. For example, a patient with a chronic disease such as diabetes may have different conditions over time. How to apply appropriate deep learning to longitudinal datasets has become a research direction.

Because of its memory function and ability to remember the information of data, recurrent neural networks such as LSTM and GRU have become the main methods to process longitudinal datasets. In general, some researchers use joint models to predict disease trajectories over time using longitudinal and time–event datasets. However, it can also reduce the accuracy of the model by applying it to large datasets. To solve this problem, Lim and van der Schaar (2018) developed a joint framework using recurrent neural networks to capture the relationship between disease trajectories using shared representations. Lee et al. (2020) combined recurrent neural networks with the attention mechanism to learn the complex relationship between the trajectory and survival probability and learn the distribution of time–event without making any assumptions about the stochastic models of longitudinal and time–event processes. Beaulieu-Jones et al. (2018) used autoencoder to represent patient care events in low dimensional vector space. They then used LSTM to predict survival rates from the sequence of nursing events learned. Lee et al. (2019) used various types of data related to Alzheimer disease to predict mild cognitive impairment. They proposed a model that used the deep learning method to learn task-related feature representation from data.

Table 3 shows some deep learning models used in computational medicine.

**TABLE 3:** Some deep learning models used in computational medicine.

**Domain**	**Model**	**Brief introduction**	**URL**
	Breast cancer type classification (Rakhlin et al., 2018)	A simple and effective method for the classification of hematoxylin and eosin–stained histological breast cancer images	https://github.com/alexander-rakhlin/ICIAR2018
Clinical image	DeepKnee (Tiulpin et al., 2018)	An automatic pipeline for osteoarthritis severity assessment from plain radiographs	https://github.com/MIPT-Oulu/DeepKnee
	Robotic instrument segmentation (Shvets et al., 2018)	A robotic instrument segmentation approach based on the deep learning network architecture	https://github.com/ternaus/robot-surgery-segmentation
	Segmentation of the left ventricle (Avendi et al., 2016)	An automatic segmentation approach of the left ventricle using deep learning and deformable model	https://github.com/alexattia/Medical-Image-Analysis
Electronic health record	Embeddings (Choi Y. et al., 2016)	A deep learning method that learns low-dimensional representations of concepts in medicine	https://github.com/clinicalml/embeddings
	Med2Vec (Choi Y. et al., 2016c)	A representation learning model for learning code representations and visit representations	https://github.com/mp2893/med2vec
	Doctor AI (Choi Y. et al., 2016b)	An automatic diagnosis machine that predicts medical codes	https://github.com/pckuo/doctorai
	Patient2Vec (Zhang X. et al., 2018a)	A deep learning method that learns an interpretable deep representation of longitudinal electronic health records data	https://github.com/BarnesLab/Patient2Vec
	DeepCare (Pham et al., 2016)	A deep learning model that reads electronic health record data and infers disease progression and predicts future outcome	https://github.com/trangptm/DeepCare
	GRU-D (Che et al., 2017)	Captures the informative missingness	https://github.com/fteufel/PyTorch-GRU-D
Genomics	DeepChrome (Singh et al., 2016)	A deep learning framework that learns combinatorial interactions among histone modification marks to predict the gene expression	https://github.com/QData/DeepChrome
	D-GEX (Chen Y. et al., 2016b)	A deep learning method that infers the expression of the target gene from the expression of the marker gene	https://github.com/uci-cbcl/D-GEX
	ADAGE (Tan et al., 2016)	Analysis using Denoising Autoencoders for Gene Expression	https://github.com/greenelab/adage
	AttentiveChrome (Singh et al., 2017)	A unified architecture that models and interprets dependencies among chromatin factors for controlling gene regulation	https://github.com/QData/AttentiveChrome
	GEDFN (Kong and Yu, 2018)	A deep learning classifier embedding feature graph information	https://github.com/yunchuankong/GEDFN
	CancerTypePrediction (Mostavi et al., 2020)	A model that uses gene expression inputs and predicts cancer types	https://github.com/chenlabgccri/CancerTypePrediction
	Deepnet-RBQ (Zhang et al., 2016)	A multimodal deep belief network that predicts the target sites of RNA-binding proteins	https://github.com/thucombio/deepnet-rbp
	DeepCpG (Angermueller et al., 2017)	A model for predicting the methylation state of CpG dinucleotides in multiple cells	https://github.com/PMBio/deepcpg
	SPEID (Singh et al., 2019)	A deep neural network for predicting enhancer–promoter interactions from sequence data	https://github.com/ma-compbio/SPEID
	Xpresso (Agarwal and Shendure, 2020)	Deep learning models for predicting gene expression levels from genomic sequence	https://github.com/vagarwal87/Xpresso
	Basset (Kelley et al., 2016)	A tool for learning highly accurate models of DNA sequence activity	https://github.com/davek44/Basset
	Integrative deep models for alternative splicing (Jha et al., 2017)	Deep learning models for alternative splicing	https://majiq.biociphers.org/jha_et_al_2017/
	ExPecto (Zhou et al., 2018)	A deep learning framework for predicting expression effects of human genome variants *ab initio* from sequence	https://github.com/FunctionLab/ExPecto
	Gene2vec (Zou et al., 2019)	A deep learning neural embedding for prediction of mammalian N6-methyladenosine sites	http://server.malab.cn/Gene2vec/
	CNNC (Yuan and Bar-Joseph, 2019)	A deep learning method for inferring gene relationships from single-cell expression data	https://github.com/xiaoyeye/CNNC
Drug development	DeepDTIs (Wen et al., 2017)	A deep belief network for predicting the interaction between drugs and targets	https://github.com/Bjoux2/DeepDTIs
	DeepDTA (Öztürk et al., 2018)	The convolutional neural networks for predicting the binding affinity value of drug–target pairs	https://github.com/hkmztrk/DeepDTA
	deepDTnet (Zeng et al., 2020)	A deep learning method for predicting drug–target interactions.	https://github.com/ChengF-Lab/deepDTnet
	MLP (Aliper et al., 2016)	A deep learning model that predicts pharmacological properties of drugs and drug repurposing	https://github.com/alvarouc/mlp
	DeepSynergy (Preuer et al., 2018)	A deep learning approach for predicting the synergy of drug combinations	http://www.bioinf.jku.at/software/DeepSynergy/
	deepDR (Zeng et al., 2019)	A deep learning approach for inferring new drug–disease relationships for in silicon drug repurposing	https://github.com/ChengF-Lab/deepDR
	DeepCPI (Wan et al., 2019)	A deep learning framework for large-scale *in silico* drug screening	https://github.com/FangpingWan/DeepCPI
	Drug-Combo-Generator (Karimi et al., 2020)	Deep generative models for drug combination generation	https://github.com/Shen-Lab/Drug-Combo-Generator

## Problems and Challenges

Although deep learning has achieved better results than machine learning in the medical and health field, there are still some challenges and problems. Here, we highlight the following problems and challenges and explore some solutions to them.

### Data Insufficiency

Deep learning is a data-driven approach. Generally, there are many parameters in the neural networks, which need to be learned, updated, and optimized from the data. With the advent of the era of big data, sufficient data provide a data basis for the development of deep learning. Therefore, deep learning has achieved great success in many data fields, such as image recognition, natural language processing, and computer vision. However, in the field of health care, medical datasets are usually limited and biased. Because the number of health samples is far more than the number of disease cases, or the number of images in each category is uneven, the application of deep learning in this field is difficult.

Insufficient data will limit the parameter optimization of deep learning and lead to the problem of overfitting. The performance of the learned model on the training set is good, but the performance on the data that have never been seen is very poor. The generalization ability of the model is poor. Usually, dropout and regularization are two common methods to solve overfitting. Besides, an increasing dataset is also a common means to suppress overfitting. In clinical imaging, data enhancement is a method to expand datasets. The data enhancement is to use translation, rotation, clipping, scaling, changing contrast, and other methods to generate new images. For example, Rakhlin et al. (2018) used data enhancement on a small breast cancer histological image dataset to improve the robustness of the model.

Transfer learning is also an effective way to solve the problem of insufficient data. Transfer learning means that the model first learns on a task with sufficient data and then applies the learned model to another related task and then fine-tunes the model parameters. For example, it can transfer the neural network model trained on a large number of natural images to the task of small sample medical images. Many experiments have proven that transfer learning is an effective method. For example, Shin et al. (2016) have proven that the transfer learning using datasets from large-scale annotated natural image datasets (ImageNet) to computer-aided diagnosis datasets is beneficial in experiments.

In recent years, multimodal learning has become a trend to solve this problem. Multimodal learning can learn different types of data simultaneously, which uses different types of data as input, such as electronic health records, medical images, and genomic data. According to the characteristics of different types of data, different models are developed. Finally, the information is integrated to provide the model ability. For example, Dai et al. (2018) integrated clinical reports with fundus images to detect retinal microaneurysms. Combining expert domain knowledge and image information solves the problem of training neural networks under extremely unbalanced data distribution.

### Model Interpretability

Deep learning is often considered as a “black box” because of its lack of explaining ability. In some areas, such as image recognition, the lack of interpretability may not be a big problem. Still, in the field of health care, the interpretability of models is very important. Because if a model can provide sufficient and reliable information, doctors will trust the results of the model and make correct and appropriate decisions; at the same time, an interpretable model can also provide a comprehensive understanding for patients.

Some researchers have been exploring the interpretive problems of neural networks. Lanchantin et al. (2016) proposed an optimization strategy to extract features or patterns to visualize gene sequence classification. In the form of statistical physics, Finnegan and Song (2017) extracted and interpreted the features of sequences learned by the network. Choi E. et al., 2016 used the attention model to detect the part of electronic health records that affected the predictive ability of the model.

### Privacy and Ethical Issues

Data privacy is an important aspect in the medical and health field. The improper use, misuse, and even abuse of patient data will bring disastrous consequences. As we know, deep learning training requires a large number of representative datasets. These datasets are very valuable, but they can be very sensitive.

At present, in computational medicine, many researchers have developed and publicly shared their deep learning models for others to use. There are many parameters in the deep learning model, which may contain sensitive information of data. Some people with ulterior motives may design some ways carefully to attack deep learning models. They can infer these parameters from the deep learning model and even infer the sensitive information in the dataset, thus violating the privacy of the model and patients. Phong et al. (2018) pointed out that even a small part of the gradient stored on cloud services can cause information leakage of local data. So, they use a homomorphic encryption mechanism to solve the problem of information leakage. Hitaj et al. (2017) pointed out that the generation of countermeasures network can recover the information in the data. Shokri and Shmatikov (2015) pointed out that the parameters of neural networks might leak the information of the training set when training neural networks.

In recent years, some researchers have explored the safety of deep learning. For example, Abadi et al. (2016) proposed a differential privacy random gradient descent algorithm by combining deep learning with differential privacy. By developing new technologies, they improved the computational efficiency of differential privacy in deep learning. These technologies include an efficient gradient algorithm, a differential privacy projection in the input layer, and so on. Their method is universal and can be applied to many classical optimization algorithms to solve the privacy problem in deep learning. Lecuyer et al. (2019) proposed PixelDP, which is the first certified defense and can be extended to large networks and datasets and widely applied to any type of deep learning model. They did a quantitative analysis for the robustness of the deep neural network to counter samples and achieved a good defense effect.

Due to the explosive growth of data, some users will put their data on the cloud, which brings challenges for deep learning to cloud computing the data provided by different data owners. In order to solve the privacy problem of collaborative deep learning in cloud computing, Li et al. (2017) provided two schemes to protect the privacy of deep learning. One is the basic scheme, which is based on multi-key fully homomorphic encryption; the other is the advanced scheme, which combines double decryption mechanism with fully homomorphic encryption. They have proven that the two schemes are secure in maintaining multi-key privacy on encrypted data. Yuan et al. (2019) used the differential privacy method to add Gaussian noise to the shared parameters to solve the problem of privacy leakage caused by shared parameters.

Patient data contains very sensitive information, which brings challenges to the application and development of deep learning in the field of computational medicine, resulting in a vicious circle. A hospital or researcher has a huge amount of patient privacy information, once the information is leaked, it will cause incalculable loss and bad influence. On the contrary, hospitals and researchers are unwilling to disclose their patient information and data because they are worried about the risk of data leakage, which will lead to the problem that deep learning cannot take advantage of large-scale data.

Because of the privacy of patients’ data, the sharing of medical data has become a very complex and difficult problem. It involves not only moral and legal issues but also technical issues. Some countries have adopted laws and regulations to regulate the use of user sensitive information by organizations. For example, on May 25, 2018, the European Union(EU) issued a very strict privacy protection regulation, the General Data Protection Regulation (GDPR). The application scope of the regulation is very wide. Any organization dealing with the data of EU users must comply with the regulation. Anyone who violates the regulation will face a great degree of punishment. GDPR defines user data as personal identifier information. As long as the data can locate users, it is considered as personal identifier information, and this data must be strictly protected. The promulgation of GDPR is a start. With the increasing importance of personal privacy today, other countries or regions will also issue similar policies to protect people’s privacy. These policies will bring a profound impact on the field of artificial intelligence driven by big data.

How to make full use of the advantages of big data to promote the development of deep learning under the premise of protecting patients’ privacy data is a problem that must be considered in the application of deep learning, that is, artificial intelligence in the field of computational medicine.

### Heterogeneity

The data in the field of health care are full of heterogeneity. There are both unstructured data and structured data. In addition, the data in the field of health care are noisy, high-dimensional, and of low quality.

Because of the existence of these heterogeneous data, it is difficult to find a suitable deep learning model. We know that the input data of neural networks must be processed and converted into a numerical value. How to properly preprocess the structured and unstructured biomedical data is a problem that researchers should first consider when training the neural networks. Therefore, processing these data is also one of the challenges faced by applying deep learning in the medical field. Some researchers have explored the processing of medical imaging datasets. For example, Li et al. (2020) intensively annotated medical images based on anatomical and pathological features. Their research expanded the label of the dataset and effectively solved the problem of small data sample size. The results show that the algorithms trained on the densely annotated medical imaging datasets they used have significantly higher diagnostic accuracy.

## Conclusion

We surveyed the application of deep learning in computational medicine such as clinical imaging, electronic health record, genomics, and drug development. Using deep learning to process big biomedical data can mine the information in the data to better provide guidance for doctors and improve the level of medical health. At the same time, this article also points out the problems and challenges in the application of deep learning in computational medicine. It provides a reference and way to improve the application of deep learning in the medical and health field in the future.

## Author Contributions

XL conceived and designed the survey. SY analyzed the data and contributed reagents, materials, and analysis tools. All authors wrote the manuscript.

## Conflict of Interest

The authors declare that the research was conducted in the absence of any commercial or financial relationships that could be construed as a potential conflict of interest.
